# Restriction of Zika virus infection and transmission in *Aedes aegypti* mediated by an insect-specific flavivirus

**DOI:** 10.1038/s41426-018-0180-4

**Published:** 2018-11-15

**Authors:** Hannah Romo, Joan L. Kenney, Bradley J. Blitvich, Aaron C. Brault

**Affiliations:** 1 Division of Vector-Borne Diseases, National Center for Emerging Zoonotic Infectious Diseases, Centers for Disease Control and Prevention, Fort Collins, CO 80521 USA; 2 Department of Veterinary Microbiology and Preventive Medicine, College of Veterinary Medicine, Iowa State University, Ames, IA 50011 USA

## Abstract

Previous studies demonstrated an insect-specific flavivirus, Nhumirim virus (NHUV), can suppress growth of West Nile virus (WNV) and decrease transmission rates in NHUV/WNV co-inoculated *Culex quinquefasciatus*. To assess whether NHUV might interfere with transmission of other medically important flaviviruses, the ability of NHUV to suppress viral growth of Zika virus (ZIKV) and dengue-2 virus (DENV-2) was assessed in *Aedes albopictus* cells. Significant reductions in ZIKV (100,000-fold) and DENV-2 (10,000-fold) were observed in either cells concurrently inoculated with NHUV or pre-inoculated with NHUV. In contrast, only a transient 10-fold titer reduction was observed with an alphavirus, chikungunya virus. Additionally, restricted in vitro mosquito growth of ZIKV was associated with lowered levels of intracellular ZIKV RNA in NHUV co-inoculated cultures. To assess whether NHUV could modulate vector competence for ZIKV, NHUV-inoculated *Aedes aegypti* were orally exposed to ZIKV. NHUV-inoculated mosquitoes demonstrated significantly lower ZIKV infection rates (18%) compared to NHUV unexposed mosquitoes (51%) (*p* < 0.002). Similarly, lower ZIKV transmission rates were observed for NHUV/ZIKV dually intrathoracically inoculated mosquitoes (41%) compared to ZIKV only inoculated mosquitoes (78%) (*p* < 0.0001), suggesting that NHUV can interfere with both midgut infection and salivary gland infection of ZIKV in *Ae. aegypti*. These results indicate NHUV could be utilized to model superinfection exclusion mechanism(s) and to study the potential for the mosquito virome to impact transmission of medically important flaviviruses.

## Introduction

The genus *Flavivirus* comprises a diverse group of viruses that phylogenetically cluster based on host or vector usage and includes insect-specific flaviviruses (ISFs), dual-host tick-borne flaviviruses, dual-host mosquito-borne flaviviruses (MBFVs), and viruses with no known vector^1^. Flaviviruses within the MBFVs group such as dengue virus (DENV 1-4), West Nile virus (WNV), yellow fever virus, Japanese encephalitis virus (JEV), and Zika virus (ZIKV) are the most recognized since they are the causative agents of disease in humans and animals worldwide^2,3^. In contrast, flaviviruses within the ISFs group grow within invertebrate vectors but are incapable of growth within vertebrates and are likely maintained primarily through vertical transmission between infected female mosquitoes and progeny^4^. The ISFs consist of two phylogenetically distinct groups of viruses, a basal lineage that forms the root of the flavivirus clade and is believed to be ancestral [hereby designated as classical insect-specific flaviviruses (cISFs)], and a group that clusters among the MBFVs for which members have presumably, based on codon/dinucleotide usage patterns present within their genomes, lost the capacity to replicate in vertebrate hosts. This latter group of ISFs have been designated as dual-host associated insect-specific flaviviruses (dISFs)^5^.

Improvements in sequencing technologies over the last decade have quickened the pace of ISF discovery. Studies designed to address the capacity of cISFs to impact the transmission of medically important flaviviruses have been performed, but results have varied. Two studies previously reported a positive association in field-derived mosquitoes between WNV and Culex flavivirus (CxFV), a cISF with a worldwide distribution^6,7^. However, another research group assessing the prevalence of WNV among field caught *Culex quinquefasciatus* mosquitoes from Georgia found no evidence supporting a positive association between WNV and CxFV^8^. Contrasting results have also been observed with CxFV in laboratory-adapted *Culex* mosquitoes where previous vertical infection with CxFV either delayed the time to transmission of WNV in *Culex pipiens* or enhanced WNV transmission in *Cx. quinquefasciatus*^9,10^. Intriguingly, a cISF isolated from *Coquillettidia xanthogaster*, Palm Creek virus (PCV), has been shown to decrease titers of WNV and Murray Valley encephalitis when *Aedes albopictus* (C6/36) cells were superinfected with PCV and also to decrease the proportion of *Cx. pipiens* capable of transmitting WNV following per oral exposure^11,12^. Together, these studies suggest that the capacity of cISFs to alter vector competence of mosquitoes for other flaviviruses is complex, could be viral and/or vector-specific, and could be modulated by several different mechanisms.

Nhumirim virus (NHUV) is a dISF that was originally isolated from a pool of *Culex chidesteri* mosquitoes in the Pantanal region of Brazil^13^. Despite its close phylogenetic placement within the dual-host mosquito-borne flaviviruses, no vertebrate cells assayed have been shown to be competent for NHUV growth. Structural elements within the 3′ untranslated region (UTR) and codon usage patterns of NHUV, similar to those of MBFVs, suggest that NHUV likely recently diverged from an ancestral mosquito-borne flavivirus^14^. The ability of NHUV to interfere with the replication of three medically important *Culex*-associated flaviviruses, Saint Louis encephalitis virus (SLEV), JEV and WNV, has previously been characterized in multiple mosquito lines. Significantly lower titers were observed for all three of the aforementioned viruses in cells previously or co-inoculated with NHUV^14^. *Cx. quinquefasciatus mosquitoes* co-infected with WNV/NHUV exhibited lower WNV transmission rates than NHUV unexposed mosquitoes, indicating that NHUV has the capacity to alter transmission phenotypes of competing flaviviruses through undetermined mechanism(s)^12,15^. Herein, the ability of NHUV to interfere with the in vitro mosquito growth/replication of two medically important *Aedes*-associated flaviviruses, ZIKV and DENV-2, as well as the transmission of ZIKV by *Aedes aegypti* was assessed.

## Results

### Inhibition of ZIKV and DENV-2 growth, but not CHIKV, by NHUV in C6/36 cells

**Fig. 1:**
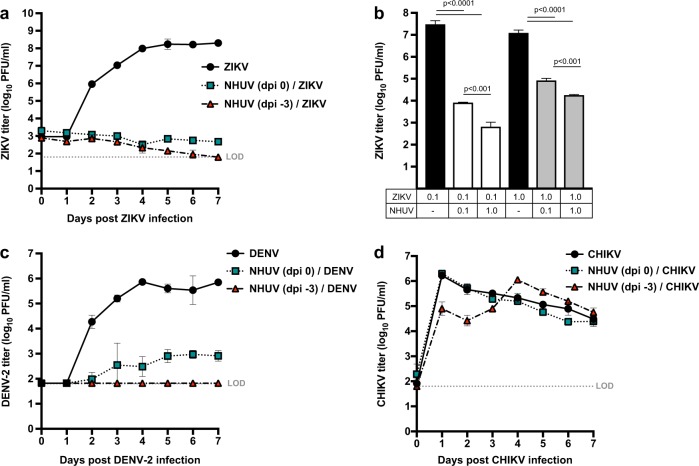
NHUV reduces viral titers of ZIKV and DENV-2, but not CHIKV, in C6/36 cells. ZIKV (**a**) titers of C6/36 cells superinfected with NHUV (MOI 5) at −3 or 0 dpi prior to ZIKV infection (MOI 0.1). **b** ZIKV titers at 7 dpi from cells co-inoculated with NHUV or ZIKV at varying MOI combinations (MOI = 0.1 or 1.0). DENV-2 (MOI 0.1) (**c**) or CHIKV (MOI 0.1) (**d**) titers of C6/36 cells superinfected with NHUV (MOI 5) at −3 or 0 dpi. Inoculations for all groups were performed in triplicate. The limit of detection (LOD) for ZIKV, DENV-2, or CHIKV was 1.8 log_10_ PFU/ml culture supernatant

Previous studies have demonstrated that NHUV suppressed the in vitro growth of WNV (1,000,000-fold), JEV (80-fold), and SLEV (15-fold) in mosquito cells pre-inoculated or co-inoculated with NHUV^14^. In order to assess whether NHUV might also interfere with other globally distributed arboviruses of medical importance transmitted by *Aedes* spp. vectors, the capacity of NHUV to suppress in vitro viral growth of ZIKV, DENV-2, and chikungunya virus (CHIKV) in C6/36 was assessed. C6/36 cells were inoculated with NHUV either concurrently or 3 days prior to inoculation with ZIKV, DENV-2, or CHIKV. A significant reduction in ZIKV titer was observed for cultures co-inoculated or previously inoculated with NHUV. ZIKV titers of NHUV/ZIKV inoculated cultures were never observed to be above those identified at the initial time point < 3.3 log_10_ (PFU/ml), indicating little to no viral growth. In contrast, a peak titer of 8.2 log_10_ (PFU/ml) for ZIKV only inoculated cultures was observed, indicating a ≥100,000-fold reduction in ZIKV peak titer (2–7 dpi, *p* < 0.0001) (Fig. 1a) between the treatment groups. Titers of ZIKV in ZIKV only inoculated cultures were significantly higher 2–7 dpi (*p* < 0.0001). No significant differences in ZIKV titers were observed between cells co-inoculated with NHUV or superinfected with NHUV three days earlier.

The aforementioned in vitro growth experiments demonstrating the suppressive effect of NHUV on ZIKV titers were performed with a NHUV multiplicity of infection (MOI) that was 50-fold higher than that of ZIKV (Fig. 1a). To determine whether NHUV could mediate a reduction in ZIKV growth with a less biased NHUV input dose, C6/36 cells were inoculated concurrently with varying ZIKV and NHUV MOIs (Fig. 1b). ZIKV titers in cultures inoculated with ZIKV at an MOI of 0.1 reached 7.4 log_10_ PFU/ml at 7 dpi. In contrast, cultures concurrently inoculated with ZIKV and NHUV at equivalent MOIs (0.1) demonstrated a 3,000-fold decrease (*p* < 0.0001) in ZIKV titer. Cultures concurrently inoculated with ZIKV (0.1) and NHUV (1.0) demonstrated a 40,000-fold decrease (*p* < 0.0001) in ZIKV titer. This 10-fold higher NHUV MOI (1.0) significantly reduced ZIKV titers (*p* < 0.001) compared to the NHUV (MOI 0.1)/ZIKV (0.1) co-inoculation group, demonstrating that the suppression of ZIKV by NHUV was dose-dependent. When cultures were co-inoculated with ZIKV at a ten-fold higher dose than NHUV, ZIKV titers were still significantly reduced 150-fold but significantly less than when inoculated at equal (1.0) MOIs (800-fold; *p* < 0.0001).

A significant reduction in DENV-2 titers were observed for C6/36 cells simultaneously inoculated with NHUV and DENV-2 (Fig. 1c). The mean peak titer for DENV-2 was only 2.9 log_10_ PFU/ml in NHUV/DENV co-inoculated cells. In contrast, the mean peak titer in DENV-2 only inoculated cultures was significantly higher [5.8 log_10_ PFU/ml (800-fold reduction)]. No detectable DENV-2 growth (≤1.8 log_10_ PFU/m) was observed for the superinfected NHUV (-3 dpi)/DENV-2 cultures at all time points, resulting in a ≥10,000-fold decrease in DENV-2 titer (Fig. 1c). To assess whether this inhibitory effect was flavivirus-specific, viral titers of an alphavirus, CHIKV, were similarly compared in cultures co- and previously inoculated with NHUV (Fig. 1d). Although significantly lower CHIKV titers were observed at one and two dpi for cultures pre-inoculated with NHUV compared to cultures not exposed to NHUV, this difference was observed to only be transient with no differences in CHIKV titers observed for co-inoculated cultures 3–7 dpi.

### NHUV restricts ZIKV RNA replication in C6/36 cells

**Fig. 2:**
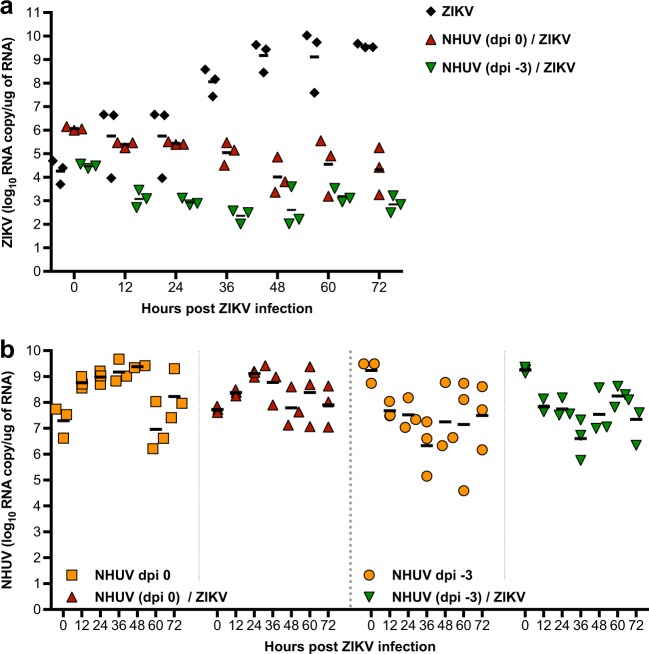
NHUV restricts intracellular replication of ZIKV in C6/36 cells. **a** Intracellular ZIKV RNA copy number in C6/36 cells superinfected with NHUV (MOI 5.0) at −3 dpi or concurrently inoculated with NHUV (MOI 5.0) and ZIKV (MOI 0.1). **b** The same NHUV/ZIKV groups assayed for ZIKV copy number were also assayed from NHUV and compared to intracellular NHUV RNA copy number in C6/36 cells inoculated with NHUV only at similar time points. For all groups, RNA was extracted from harvested cells and the copy number for NHUV or ZIKV was determined by qRT-PCR. Infections for all groups were performed in triplicate. Each point represents total extracted RNA from a single well

Superinfection exclusion between different WNVs has been shown to occur at the level of RNA replication^16^. To assess whether ZIKV RNA replication is affected by intracellular replication of NHUV, intracellular levels of NHUV or ZIKV RNA were assessed in C6/36 cells following inoculation with ZIKV or NHUV/ZIKV. For ZIKV only inoculated cultures, the level of ZIKV RNA increased over time (Fig. 2a). The highest RNA level (9.5 log_10_ ZIKV RNA copies/μg RNA) was observed at 72 h post-inoculation (hpi). In contrast, ZIKV RNA levels were significantly lower than the input at 48 hpi (*p* < 0.05) and 72 hpi (*p* < 0.05) for cultures pre-inoculated with NHUV 3 days earlier or at 36 (*p* < 0.01) and 48 hpi (*p* < 0.05) for cells co-inoculated with NHUV. ZIKV RNA levels never increased above the input level for both groups at all other time points, indicating that ZIKV RNA replication was restricted by NHUV. To examine whether ZIKV infection could reciprocally negatively impact NHUV replication, the levels of NHUV RNA were also measured in the same cultures described above for which ZIKV was quantified. NHUV RNA copy number was high in groups inoculated with NHUV only and in ZIKV co-inoculated groups (−3 dpi or 0 dpi). No statistically significant differences in NHUV RNA levels were observed between the groups, suggesting that ZIKV exposure to the C6/36 cells had no detectable effect on NHUV RNA replication (Fig. 2b).

### NHUV does not inhibit ZIKV cellular entry

**Fig. 3:**
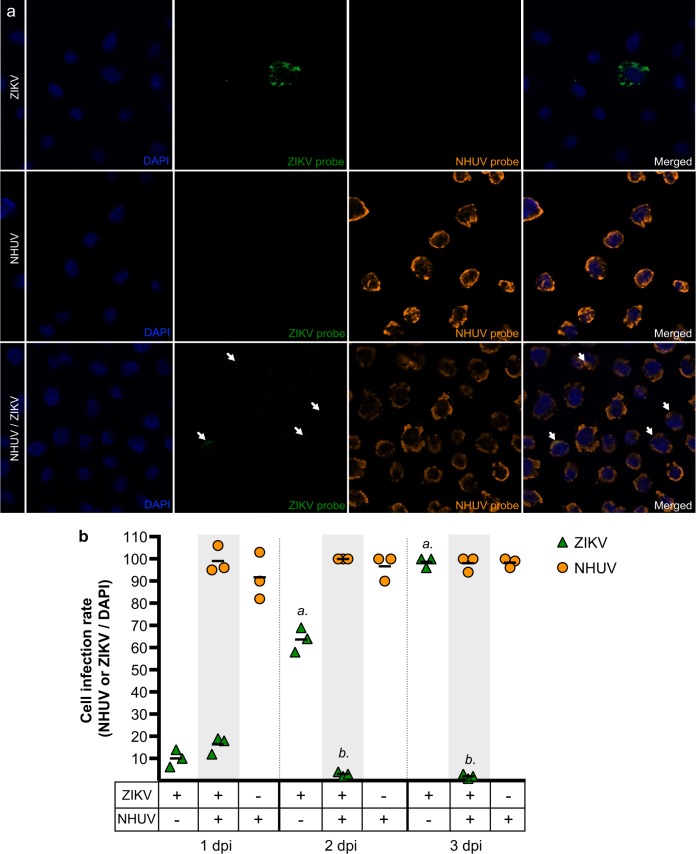
NHUV does not inhibit cellular infection of ZIKV in C6/36 cells. **a** ISH of C6/36 cells co-inoculated with ZIKV and NHUV or singly inoculated with NHUV and ZIKV and imaged at 1 dpi. **b** Quantification of DAPI stained cells that were also positive by ISH for NHUV, ZIKV, or NHUV and ZIKV RNA assessed through 1–3 dpi. The nuclei were stained by DAPI. Comparisons where the *p*-values are lower than 0.05 were observed for ZIKV positive cells in groups inoculated with ZIKV only or NHUV/ZIKV and is denoted by *a* and *b*, where *a* is significantly different from *b*

Co-infection or previous infection with NHUV significantly altered both in vitro intracellular RNA replication and growth of ZIKV; however, no significant differences in viral growth or intracellular replication of ZIKV were observed between the NHUV infection timing groups. In order to determine if NHUV-mediated inhibition of ZIKV was related to cellular entry, highly sensitive in situ hybridization (ISH) for NHUV and ZIKV RNA was performed on NHUV (MOI 5.0) and ZIKV (MOI 0.1) co-inoculated C6/36 cells (Fig. 3a). At 1 dpi, ZIKV or NHUV RNA was visible in cells inoculated with ZIKV or NHUV only, respectively. Interestingly, in NHUV/ZIKV co-inoculated cells, ZIKV and NHUV RNA were found in the same cells, suggesting that NHUV-mediated inhibition of ZIKV was not occurring at the level of viral entry. The presence of NHUV and ZIKV RNA in cells was quantified from 1 to 3 dpi (Fig. 3b). No differences in the number of cells containing ZIKV RNA between the ZIKV only and NHUV/ ZIKV inoculated groups were observed at 1 dpi. The percentage of cells exhibiting detectable ZIKV RNA in ZIKV only inoculated cultures increased over time, reaching 100% by 3 dpi. In contrast, the percentage of cells with detectable ZIKV RNA decreased for NHUV/ZIKV co-inoculated groups on 2 dpi (*p* < 0.0001) and 3 dpi (p < 0.0001) in comparison to ZIKV only inoculated cultures. For groups inoculated with NHUV or NHUV/ZIKV, the presence of NHUV RNA was nearly 100% at 1 dpi and remained at that level through 3 dpi. No differences in the percentage of NHUV RNA observed in NHUV only inoculated cultures or NHUV/ZIKV co-inoculated cultures were observed at any time points (Fig. 3b).

### ZIKV oral infection rates are reduced in *Ae. aegypti* intrathoracically inoculated with NHUV

**Fig. 4:**
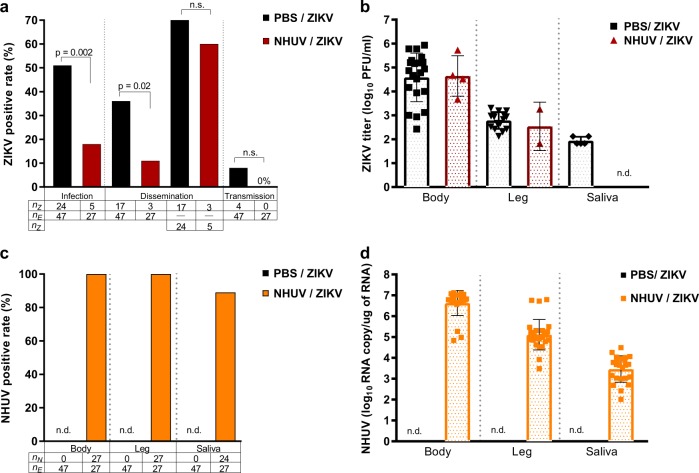
Reduced ZIKV infection rates observed in *Ae. aegypti* previously inoculated with NHUV. 2–4 day-old *Ae. aegypti* were IT inoculated with NHUV or PBS and per orally exposed to a ZIKV infectious blood meal at 6 days post IT inoculation. **a** Infection, dissemination, and transmission rates for ZIKV. Infection rates were determined as the number of ZIKV positive bodies (*n*_Z_) as a function of blood fed mosquitoes (*n*_e_). Dissemination rates were calculated as the number of ZIKV positive legs (*n*_z_) per exposed mosquitoes (*n*_e_) as well as the number of ZIKV positive legs from ZIKV positive bodies. Transmission rates were calculated as the percentage of ZIKV exposed mosquitoes (*n*_e_) that were also positive for virus in saliva (*n*_z_). **b** ZIKV titers for bodies, legs and salivary expectorants. **c** Percent of bodies, legs, and salivary expectorants positive for NHUV RNA. The percentage of ZIKV positive bodies determined as the number of NHUV RNA positive bodies (*n*_N_) as a function of inoculated mosquitoes (*n*_E_). The percent of infected legs was calculated as the number of NHUV RNA positive legs (*n*_N_) as a function of inoculated mosquitoes (*n*_E_). The percent of positive saliva expectorants was calculated as the number of NHUV-inoculated mosquitoes (*n*_N_) that were also positive for NHUV RNA in saliva (*n*_E_). Not detected (n.d.) **d** NHUV RNA copy number detected for bodies, legs, and salivary expectorants. Not detected (n.d.)

To model a superinfection scenario, *Ae. aegypti* were intrathoracically (IT) inoculated with NHUV and held for 6 days in order to establish a productive NHUV infection. Mosquitoes were subsequently offered a ZIKV infectious blood meal and mosquito infection, dissemination, and transmission rates were assessed after a 14-day incubation period. The ZIKV infection rate of mosquitoes that had been inoculated with NHUV prior to ZIKV oral exposure was significantly lower compared to the rate for mosquitoes that had been previously inoculated with phosphate buffered saline (PBS) solution. Fifty-one percent of PBS-inoculated *Ae. aegypti* were observed to be infected with ZIKV compared to only 18% of mosquitoes that had been previously inoculated with NHUV prior to ZIKV per oral exposure (*p* = 0.002) (Fig. 4a). When dissemination was calculated with the denominator as the number of orally exposed mosquitoes, a significant difference between NHUV and control mosquito groups was observed (*p* = 0.02); with dissemination rates for the PBS-IT/ZIKV group at 36% and dissemination for NHUV-IT/ZIKV at 11%. However, this was a function of the significant differences in infection rate between the groups as evidenced by the loss of significance for dissemination rates when calculated with the denominator being the number of ZIKV-infected mosquito bodies. A low ZIKV transmission rate of 8% was observed for the mosquitoes in the PBS-IT/ZIKV group. While no ZIKV transmission was observed for the NHUV-IT/ZIKV exposed mosquitoes, this was not statistically significant given the low rate of transmission in the control group. The titers of ZIKV in the bodies and legs for the PBS-IT/ZIKV and NHUV-IT/ZIKV groups were not statistically significantly different (Fig. 4b). A 100% NHUV RNA positivity rate for mosquito bodies and legs was observed in mosquitoes IT inoculated with NHUV and subsequently orally exposed to ZIKV. NHUV RNA was also detected in 89% of salivary expectorants from these mosquitoes (Fig. 4c). The mean NHUV RNA loads observed in bodies, legs, and expectorants were 6.6, 5.0, and 3.5 log_10_ RNA copies per body, leg, and expectorant, respectively (Fig. 4d).

### NHUV prevents ZIKV transmission in *Ae. aegypti* following co-infection

**Fig. 5:**
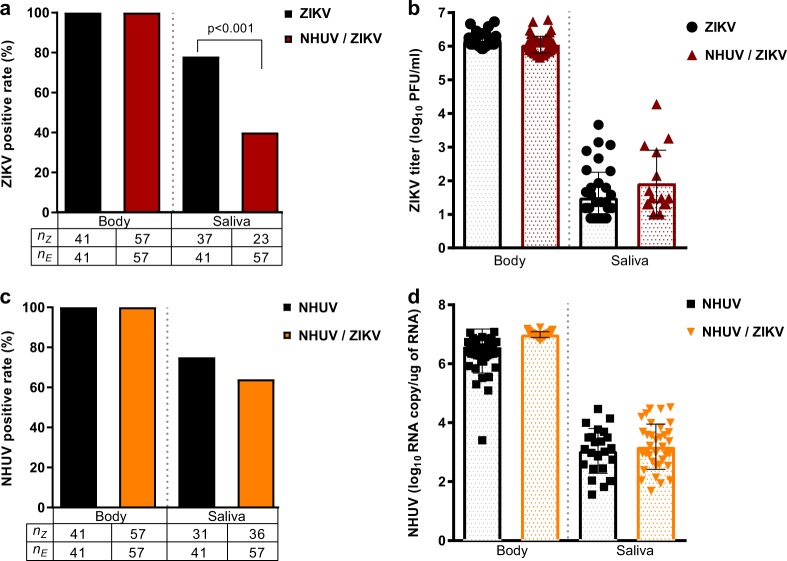
NHUV infection reduces ZIKV transmission in *Ae. aegypti*. *Ae. aegypti* IT inoculated with NHUV, ZIKV, or NHUV and ZIKV. **a** Percent of bodies positive for ZIKV and percent of saliva expectorants positive for ZIKV for mosquitoes IT inoculated with ZIKV or NHUV and ZIKV. **b** ZIKV titers of mosquito bodies or salivary expectorants for mosquitoes IT inoculated with ZIKV or NHUV and ZIKV. **c** NHUV RNA detection percentages in bodies and saliva expectorants of *Ae. aegypti* IT inoculated with NHUV or NHUV and ZIKV by NHUV-specific qRT-PCR. **d** NHUV RNA copy number of mosquito bodies and salivary expectorants

Previous studies with NHUV, initially isolated from *Cx. chidesteri*, reported the utility of NHUV to block WNV transmission in *Cx. quinquefasciatus* IT inoculated with both NHUV and WNV^14,15^. In the aforementioned experiments (Fig. 4), insufficient ZIKV transmission rates in the control group were observed to assess the capacity for NHUV to restrict ZIKV transmission. To assess transmission rates, *Ae. aegypti* were IT inoculated with NHUV, ZIKV, or NHUV and ZIKV and the presence of NHUV RNA or ZIKV were assessed in bodies and expectorants 7 days later. Infection rates for ZIKV were 100% for ZIKV and NHUV/ZIKV IT inoculated groups (Fig. 5a). In contrast, the ability of mosquitoes to transmit ZIKV was significantly reduced in NHUV/ZIKV co-inoculated mosquitoes compared to ZIKV only inoculated mosquitoes (*p* < 0.001). At 7 days post IT inoculation, 81% of mosquitoes had detectable ZIKV in expectorants in the ZIKV inoculation group as compared to only 41% for the NHUV/ZIKV inoculated group. Body and salivary expectorant titers of ZIKV from the ZIKV and NHUV/ZIKV IT inoculated groups, as determined by plaque assay, did not differ between the two groups (Fig. 5b). Similar to results observed for ZIKV infection, the detection of NHUV RNA was observed in 100% of NHUV only or NHUV/ZIKV IT inoculated mosquitoes (Fig. 5c). No statistically significant differences between the rates of NHUV RNA detection in salivary expectorants from NHUV only versus NHUV/ZIKV IT inoculation groups were observed. Furthermore, the NHUV RNA copy numbers for mosquitoes IT inoculated with NHUV only or NHUV/ZIKV were statistically indistinguishable, ranging from 6 to 7 log_10_ NHUV RNA copies for bodies, and 2–4 log_10_ NHUV RNA copies for salivary expectorants (Fig. 5d).

### NHUV RNA is detected in the salivary gland of *Ae. aegypti*

**Fig. 6:**
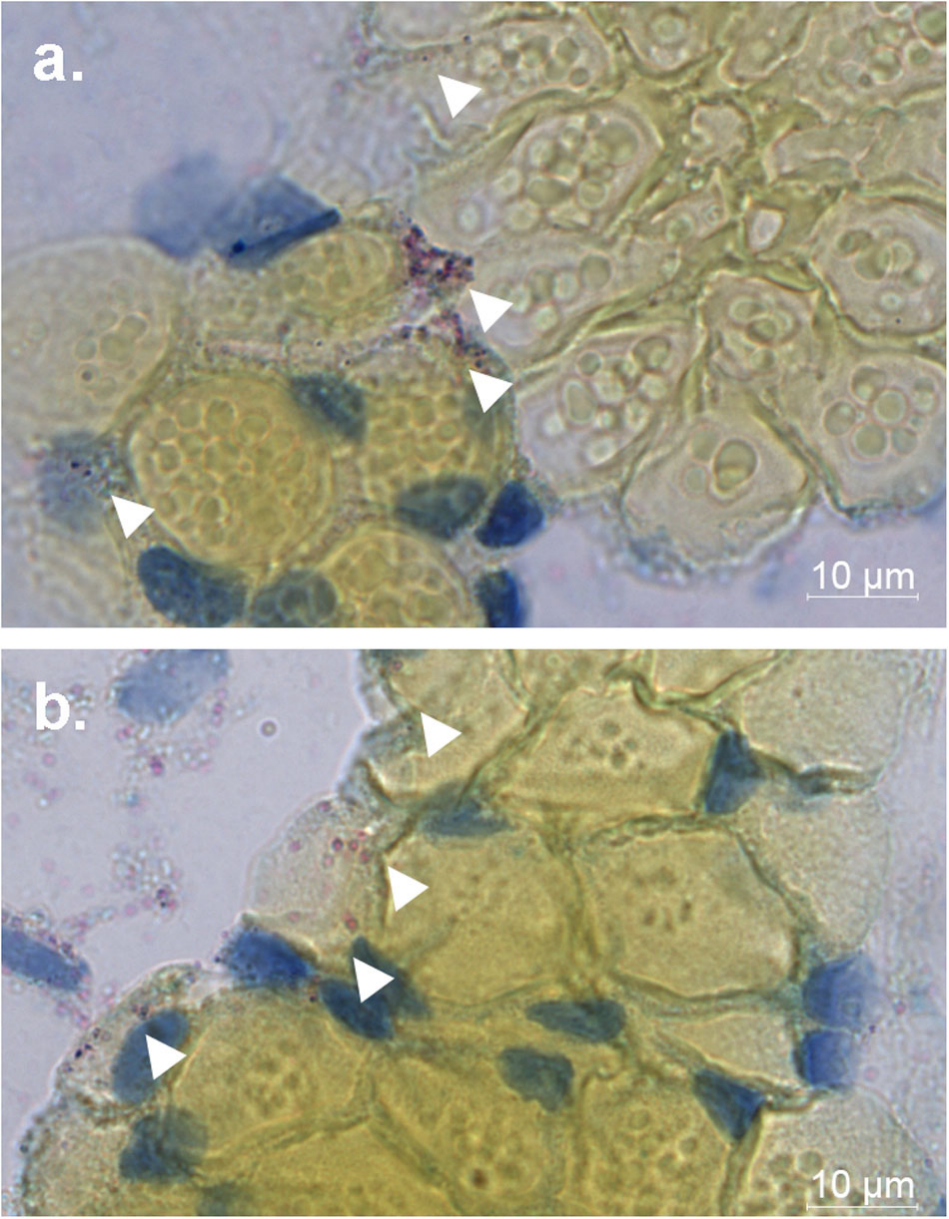
NHUV in salivary gland of *Ae. aegypti*. Chromogenic in situ hybridization (ISH) detection of NHUV (red) in dorsal (**a**) and longitudinal (**b**) salivary gland sections of *Ae. aegypti* females IT inoculated with NHUV. Counterstained with Gills hematoxylin

NHUV RNA was detected in the salivary expectorant of NHUV and NHUV/ZIKV IT inoculated mosquitoes (Fig. 6). To determine whether salivary glands could become infected with NHUV, NHUV IT inoculated mosquitoes were fixed in paraformaldehyde, thin sectioned, and stained by in situ hybridization for NHUV using a RNA probe specific for NHUV. NHUV RNA was detected in both dorsal and longitudinal sections of the salivary glands, demonstrating that NHUV has the capacity to infect salivary glands of *Ae. aegypti*.

## Discussion

Earlier in vitro studies demonstrated that NHUV reduced WNV (1,000,000-fold), JEV (80-fold), and SLEV (15-fold) titers in C6/36 cells^14^. Herein, similar in vitro experiments demonstrated that NHUV pre-infection also was capable of suppressing ZIKV (100,000-fold) and DENV-2 (10,000) growth in C6/36 cells. Whereas ZIKV demonstrated similar suppression of titers in groups either co-inoculated or superinfected with NHUV, a slightly muted suppressive effect for DENV-2 was observed in co-inoculated C6/36 cells versus cultures that were superinfected with NHUV. Similarly, SLEV and JEV, viruses that grow to lower titers and at slower rates in C6/36 cells than WNV, proportionally grew better than WNV in C6/36 cells co-inoculated with NHUV. The reason(s) for such disparate suppressive effects of NHUV for the other flaviviruses have not been elucidated but are likely related to sequence differences as well as the overall kinetics of growth and the inherent growth capacity of the viruses in C6/36 cells.

Superinfection exclusion is the capacity of a cell infected with one virus to become resistant to further infection with a secondary homologous or heterologous virus^16–18^. Initial studies illustrating the superinfection exclusion principal in arthropod cells were conducted with Sindbis virus and indicated the superinfection exclusion of alternative, distantly related alphavirus growth in C6/36 cells. In contrast, no detectible inhibition of the growth of a bunyavirus or a flavivirus was observed, suggesting that the relatedness of superinfecting viruses could be critical for this effect^19–21^. Dengue virus superinfection studies in mosquito cells demonstrated that the exclusion effect was not complete between DENV-2 and DENV-4, the effects were asymmetric and that greater suppression was observed with longer pre-infection time periods^21^. Previous studies have also demonstrated that cells expressing a WNV replicon could induce homologous and heterologous flaviviral superinfection exclusion but that no inhibition was observed for an alphavirus or a rhabdovirus^16^. The incomplete capacity of more distantly related flaviviruses to suppress superinfection of heterologous flaviviruses is illustrated by the relatively low efficiency of cISFs comprising the genetically distinct basal clade of the flaviviral phylogeny to interfere with dual-host flaviviral growth in mosquito cells. For instance, the cISFs, CxFV and PCV, have been shown to reduce WNV titers approximately 10-fold in co-inoculated C6/36 cells^9,11^. In contrast to CxFV and PCV that share only approximately 40% nucleotide identity with WNV, NHUV shares ~56% nucleotide identity with WNV and has a codon usage more similar to that of WNV, likely resulting from the recent loss of the dISFs’ ancestral vertebrate infection phenotype^14^. In the study presented herein, viral titers for CHIKV, an unrelated alphavirus, were slightly reduced and the reduction transiently observed only in cultures that were superinfected with NHUV three days prior to CHIKV infection. The capacity of NHUV, an insect-specific flavivirus, to interfere with viral growth may be related to sequence similarity of the secondary infecting virus, having a modulatory effect only on other closely related flaviviruses. These results suggest that superinfection exclusion is a virus-specific event that occurs between closely related viruses needing to compete for similar host cell resources rather than just non-specific antiviral responses mounted in the host cell^16–18^.

Superinfection exclusion can constrain different steps of the life cycle of a secondary infecting virus including at the points of viral entry, replication, or viral egress^12^. Studies presented herein demonstrated the presence of ZIKV RNA in mosquito cells concurrently inoculated with NHUV, indicating that NHUV likely did not impede ZIKV entry in a NHUV/ZIKV co-inoculation scenario. In contrast, a significant decrease in the intracellular levels of ZIKV RNA from cells co-inoculated with NHUV and ZIKV was observed, indicating that superinfection exclusion of ZIKV was occurring at the level of RNA replication. This finding was in agreement with studies performed in cells expressing WNV replicons in which inhibition of RNA replication was implicated as the principal barrier to the establishment of superinfection with other WNVs^16^. These findings do not exclude the possibility that NHUV superinfection exclusion may be also occurring for ZIKV at the level of translation or that entry may be inhibited in cells already harboring an established NHUV infection and future studies to address this possibility should be examined.

Few studies have examined the potential of ISFs (cISFs/ dISFs) to modulate the transmission of competing flaviviruses in mosquitoes. In the study presented here, a lower oral infection rate with ZIKV was observed for mosquitoes previously inoculated with NHUV as compared to sham inoculated mosquitoes, suggesting that an ISF-induced midgut infection barrier for ZIKV was potentiated by NHUV in *Ae. aegypti*. Similarly, PCV infection of *Culex annulirostris* mosquitoes has been shown to significantly reduce oral infection rates with WNV^12^. IT inoculated *Cx. annulirostris* were found to exhibit localized PCV in midgut epithelial cells, the first site within the mosquito susceptible to viral infection after oral exposure^12^. Previous studies by Smith et al.^22^, with Venezuelan equine encephalitis virus, an alphavirus, demonstrated that only a limited number of midgut epithelial cells were susceptible to infection. Although not directly assessed in these studies, it is possible that an established NHUV infection in the midgut of *Ae. aegypti* could exclude the secondary infection of a flavivirus if they are similarly limited to the number of susceptible midgut epithelial cells. It is also possible that ISFs may have a limited capacity to infect midgut epithelial cells and that infection could be restricted to the basolateral surface of the cell resulting from IT inoculation. For instance, *Cx. annulirostris* were completely refractory to oral exposure with PCV but midgut epithelial cells of PCV IT inoculated mosquitoes were infected^12^. Mosquito immune activation from IT inoculation could have resulted in a lower ZIKV oral infection rate; however, the ZIKV infection rate was directly compared to that of control IT inoculated which did not differ significantly from non-inoculated mosquitoes, indicating this was unlikely to have contributed to these findings. Despite prior infection with NHUV, a small subset of *Ae. aegypti* (18%) were infected with ZIKV. The ability of ZIKV to establish a productive infection in a limited number of NHUV positive mosquitoes may be reflective of the incomplete infection of midgut epithelial cells with NHUV. Oral exposure of these mosquitoes occurred at 6 days post NHUV-inoculation and it is possible that additional time for cell-to-cell NHUV infection in the midgut mucosa could have afforded increased refractoriness to ZIKV oral infection. Refractoriness could be afforded by activation of immune pathways in the mosquito induced by NHUV which could be variable between some mosquitoes, resulting in different efficiencies for inhibition. Studies to examine the tissue tropism of NHUV in midgut epithelial cells, immune activation pathways induced by NHUV, and how infection patterns might interfere with challenge by a heterologous flavivirus will need to be undertaken to address these questions.

A significant reduction in the ZIKV oral infection rate was observed in NHUV IT inoculated mosquitoes and for ZIKV transmission in mosquitoes that were co-IT inoculated suggesting that, in addition to blocking midgut infection, NHUV also has the capacity to impede salivary gland infection and/or expectoration of ZIKV. Similarly, Hall-Mendelin et al.^12^, demonstrated that PCV and WNV co-IT inoculated mosquitoes exhibited a significantly lower WNV transmission rate. Together, these results indicate that some ISFs may have the potential to elicit superinfection exclusion mechanisms within multiple anatomical compartments of a mosquito.

NHUV was originally isolated from *Cx. chidesteri* mosquitoes collected on a cattle ranch in the Pantanal region of Brazil; however, no data is currently available on the field infection rates of these mosquitoes or other potential vectors in Brazil or elsewhere^13^. Nevertheless, many additional closely related dISFs have been isolated in other regions of Latin America, Europe, Africa, and Asia^22–26^. NHUV, despite having been isolated from a *Culex* species, clearly demonstrates the capacity to grow within *Ae*. *aegypti*, *Cx. pipiens*, and *Cx. quinquefasciatus* mosquitoes^14,15^. As such, it is possible that some dISFs could have a wide vector range. However, since dISFs are believed to be maintained primarily through vertical transmission, a low vertical transmission efficiency of NHUV in either *Aedes* or *Culex* mosquitoes could represent a host restrictive barrier to maintenance. Further studies are needed to characterize the host range and vertical transmission potential of NHUV and other dISFs in multiple *Culex* and *Aedes* species to fully assess the potential impact of these viruses on transmission of medically important flaviviruses.

While a significantly lower ZIKV transmission rate of NHUV/ZIKV co-inoculated mosquitoes was observed, a similar lowered proportion of mosquitoes with NHUV RNA in salivary expectorants was not identified. Furthermore, the inhibitory effect did not affect titers of ZIKV observed in bodies or in the expectorants of ZIKV-infected mosquitoes. As such, this effect was an all-or-nothing phenomenon, indicating the potential that NHUV infection of all cells in the salivary gland exposed to ZIKV could be a requisite for blocking transmission or that a systemic induction of an antiviral state was required to prevent transmission. NHUV RNA was identified in 60–89% of saliva expectorants of mosquitoes either inoculated with NHUV only or dually inoculated with NHUV/ZIKV. Furthermore, the presence of NHUV RNA in an expectorant of a dually inoculated mosquito was not correlated with a reduced likelihood of that mosquito to transmit ZIKV, further indicating that NHUV was present within the salivary gland of ZIKV-transmitting mosquitoes but potentially not within the cells contributing to conveyance of infectious ZIKV to the expectorant or contributing to a systemic inhibitory state within the salivary glands. Previous studies have demonstrated that CxFV could be detected in the salivary glands of infected *Cx. pipiens* mosquitoes and could be detected in the salivary expectorants of CxFV/WNV co-infected *Cx. quinquefasciatus* mosquitoes^4,10^. Prior to these results presented herein for NHUV RNA, ISF RNA had not been identified in the salivary tissues of ISF only infected mosquitoes. Since growth of ISFs is restricted to invertebrates, vertical transmission has been postulated as the most likely route of ISF (cISF and dISF) transmission. However, based on these data, attention should be paid to the potential for horizontal transmission via sugar-feeding sources^9,26–30^^,^. The exact function of NHUV in the saliva of infected *Ae. aegypti* and other questions regarding the infectiousness of NHUV in the saliva expectorant need to be further investigated.

An ISF previously shown to limit WNV transmission has been shown here to also exhibit a capacity for suppressing ZIKV oral infection and transmission^15^. NHUV suppressed mosquito in vitro growth and replication in a flavivirus-specific manner affecting viral replication. Previous  IT inoculation with NHUV impeded oral infection with ZIKV in *Ae. aegypti*. These data coupled with the finding that dually IT inoculated mosquitoes showed a significantly reduced transmission rate, indicates that NHUV infection of *Ae. aegypti* could serve to block both oral infectivity and transmissibility, potentiating a combination effect to block vector competence for ZIKV. These results indicate that NHUV could be used as a model to further determine the unknown mechanism(s) dictating superinfection exclusion between flaviviruses and indicates the utility of additional investigations into the potential of the mosquito virome to impact vector competence of mosquitoes that transmit medically important flaviviruses.

## Materials and methods

### Viruses and cells

Low passage NHUV (C6/36 passage 3 and 4) and ZIKV (strain PRVABC59, accession number KU501215.1; passage 4 and 5) were grown on *Ae. albopictus* (C6/36) cells (ATCC, Manassas, VA) and African green monkey kidney (Vero) cells (ATCC no. CCL-81), respectively. Infection for NHUV and ZIKV was performed at an MOI of 0.1 and cell culture medium was harvested following evidence of cytopathic effect (CPE). The collected supernatant was centrifuged at 5000×*g* for 10 min to remove cellular debris and aliquots were frozen at −80 °C. ZIKV was titrated by plaque assay on Vero cells. The TCID_50_ for NHUV was determined by immunofluorescence assay with the Reed-Muench method, as previously described^14^.

### Growth curves

Growth curves were performed as previously described^14^. Triplicate cultures of *Ae. albopictus* (C6/36 cells) were simultaneously seeded at the same density in 12 well plates. Confluent cells were inoculated with NHUV at an MOI of 5 at three or zero (concurrent inoculations) day(s) prior to ZIKV, DENV-2, or CHIKV infection an MOI of 0.1. Density matched C6/36 triplicate cultures were inoculated with ZIKV, DENV-2, or CHIKV only at an MOI of 0.1 in triplicate to serve a positive control. Supernatant was collected daily through 7 days post-infection and frozen at −80 °C. Samples were titrated by plaque assay on Vero cells.

### Intracellular replication profiles

*Ae. albopictus* (C6/36) cells were simultaneously seeded at the same density in 12 well plates. Confluent cells were inoculated with NHUV at an MOI of 5 at three days prior to ZIKV infection an MOI of 0.1. Cells were also inoculated in triplicate with ZIKV at an MOI of 0.1 to serve a positive control for concurrent infections or inoculated with NHUV only at an MOI of 5.0 to serve a positive control for concurrent infections for 0 dpi or pre-exposure infections for −3 dpi. Time points were collected every 12 h through 72 h post inoculation. At each time point, the supernatant from each well was removed and cells were washed 3X with PBS. Total RNA was extracted from cells using Trizol LS (Invitrogen). ZIKV and NHUV copy numbers were quantified by real-time RT-PCR.

### ZIKV and NHUV RNA quantification

ZIKV RNA was quantified by real-time RT-PCR using a ZIKV primer and probe set as previously described^31,32^. For NHUV, a primer and probe set was designed using the primer design software in Geneious (ver 3.5) with 5-Hex as the reporter dye for the probe (forward primer, 5′-GAATGGCAGTGGAGAGGAGG-3′; reverse primer, 5′-CCTTCCATTACCACGTCCGG-3′; and probe, 5′-HEXTGCGAGATGGCAGCGGCTCTGT-BGQ1-3′) A standard curve for NHUV was generated by in vitro transcription of a plasmid containing a fragment of NHUV spanning nucleotides 5064–6036 and a standard curve for ZIKV was generated by in vitro transcription as previously described^32^.

### High resolution in situ hybridization of ZIKV and NHUV RNA in C6/36 cells

*Ae. albopictus* (C6/36) cells were simultaneously seeded at the same density in a 96 well, glass bottom plate (Corning). Confluent cells were inoculated with either NHUV only at an MOI of 5, ZIKV only at an MOI of 0.1, or concurrently inoculated of NHUV (MOI 5.0) and ZIKV (0.1 MOI). In situ hybridization of inoculated cell cultures at 1, 2, or 3 dpi was performed using the ViewRNA ISH Cell Plus according to manufacturer’s protocol with the omission of the antibody staining steps. ZIKV and NHUV RNA were detected using custom RNA probes targeting conserved regions of the positive-strand RNA for each virus. Fixed cells were stained with DAPI (1:100 dilution) and immediately imaged. Images were acquired using a Zeiss LSM800 confocal microscope and ZEN software (Carl Zeiss). Large 4 × 4 tile images were collected at ×63. Higher resolution single images were also captured at ×63 optical resolution.

### Mosquitoes

Mosquitoes used in this study were derived from laboratory maintained colonies. The *Ae. aegypti* (Poza Rico) mosquitoes were originally collected as larvae in Poza Rico, Mexico in 2000^33^. Colonies were provided with 10% sucrose ad libitum and maintained in a 70% relative humidity chamber at 28 °C on a 16:8 h light: dark cycle.

### Intrathoracic inoculations with NHUV and ZIKV

Three- to five-day-old female *Ae. aegypti* were cold-anesthetized and IT inoculated with NHUV, ZIKV, or NHUV and ZIKV. In order to mimic a scenario where the viral load of NHUV is likely greater than ZIKV in NHUV vertically infected mosquitoes, IT inoculations were performed with a 10-fold higher viral titer for NHUV than ZIKV such that 10,000 PFU [0.33 μl of 7.5 log_10_ (TCID_50_)] of NHUV was used for NHUV only IT inoculated mosquitoes, 100 PFU [0.33 μl of 5.5 log_10_ (PFU/ml)] of ZIKV was used for ZIKV only IT inoculated mosquitoes, or 100 PFU [0.33 μl of 5.5 log_10_ (PFU/ml)] of ZIKV and 10,000 PFU [0.33 μl of 7.5 log_10_ (TCID_50_)] of NHUV was used for NHUV and ZIKV IT inoculated mosquitoes. NHUV viral stock was cleared of cellular debris by centrifugation and subsequently diluted 1000-fold in PBS prior to IT inoculation in mosquitoes. Mosquitoes were held in a 70% relative humidity chamber at 28 °C. At 7 days post IT inoculation, mosquitoes were permanently anesthetized with triethylamine (Flynap®; Carolina Biological Supply company, Burlington, NC) as previously described^15^. Saliva samples were collected by placing each mosquito proboscis into a capillary tube charged with Type B immersion oil (Cargille Laboratories, Cedar Grove, NJ). After a 30-min period of expectoration, the contents of the capillary tubes were collected and expelled into 200 µl of mosquito diluent [Dulbecco’s modified Eagle’s essential medium (DMEM) supplemented with 10% fetal bovine serum, penicillin (100 U/ml), streptomycin (100 mg/ml), and amphotericin B (50 μg/ml)] by centrifugation at 5000×*g* for 10 min. Mosquito bodies were placed in a 2 μl microcentrifuge tube with 500 μl of mosquito diluent and 2 copper-coated steel shot BBs (4.5 mm diameter, 0.177″ caliber) (Qiagen). Mosquito bodies were triturated for 4 min at a frequency of 26 cycles per second using a Mixer Mill 300 (Retsch, Newton, PA). Mosquito bodies and saliva samples were stored at −80 °C until further processing. Samples were titrated by plaque assay on Vero cells. RNA was extracted from bodies and saliva samples using the MagMax Viral RNA Isolation Kit (ThermoFisher, Waltham, MA) and quantified by real-time RT-PCR.

### ZIKV per oral exposure of NHUV IT inoculated mosquitoes

Two- to three-day-old female *Ae. aegypti* were IT inoculated with 10,000 PFU [0.33 μl of 7.5 log_10_ (TCID_50_)] of NHUV or sham inoculated with PBS and held in a 70% relative humidity chamber at 28 °C on a 12:12 diurnal light cycle. At 6 days post IT inoculation, mosquitoes were offered an infectious blood meal containing freshly grown virus. To generate a virus stock to use in the ZIKV infectious blood meal that not undergone a freeze-thaw cycle, Vero cells were inoculated with ZIKV at an MOI of 0.1. Supernatant from inoculated cultures was harvested at 4 dpi and without a freeze-thaw cycle, immediately diluted in defibrinated calf blood. An aliquot of the blood meal was reserved and stored at −80 °C for later back titration of ZIKV by plaque assay [back titered at 6.5 log_10_ (PFU/ml)]. Mosquitoes were deprived of sugar and water for 24 h prior to per oral exposure. Mosquitoes were offered an infectious blood meal for 1 h using a Hemotek membrane feeder (Discovery Workshops, Accrington, UK), warmed to 37 °C. Fully engorged females were separated under cold anesthesia, contained in 1-pint cartons and provided 10% sucrose *ad libitum*. Mosquitoes were housed in 70% humidity with a 12:12 diurnal light cycle for 14 days post ZIKV per oral exposure. At 14 days post exposure, mosquitoes were permanently anesthetized with triethylamine (Flynap®; Carolina Biological Supply company, Burlington, NC) as described above. Legs were removed from individual mosquitoes and placed into eppendorf tubes containing 500 μl of mosquito diluent [DMEM complete with 10% FBS, penicillin (100 U/ml), streptomycin (100 mg/ml), and amphotericin B (50 μg/ml)]. Saliva samples were collected by placing each mosquito proboscis into a capillary tube charged with Type B immersion oil (Cargille Laboratories, Cedar Grove, NJ). After a 30 min period of expectoration, the contents of the capillary tubes were expelled into 200 µl of mosquito diluent [DMEM complete with penicillin (100 U/ml), streptomycin (100 mg/ml), 10% FBS, amphotericin B (50 μg/ml)] by centrifugation at 5000×*g* for 10 min. Mosquito bodies were placed in a 2 μl microcentrifuge tube with 500 μl of mosquito diluent and 2 copper-coated steel shot BBs (4.5 mm diameter, 0.177” caliber) (Qiagen Hilden, Germany). Mosquito bodies and mosquito legs were triturated for 4 min at a frequency of 26 cycles per second using a Mixer Mill 300 (Retsch, Newton, PA). Mosquito bodies, legs, and saliva samples were stored at −80 °C until further processing. Samples were titered by plaque assay on Vero cells. RNA was extracted from bodies and saliva samples using the MagMax Viral RNA Isolation Kit (ThermoFisher, Waltham, MA) and quantified by real-time RT-PCR.

### Chromogenic in situ hybridization of NHUV RNA in salivary glands of *Ae. aegypti*

Five- to seven-day-old female *Ae. aegypti* were IT inoculated with 10,000 PFU [0.33 μl of 7.5 log_10_ (TCID_50_)] of NHUV and held in a 70% relative humidity chamber at 28 °C on a 12:12 diurnal light cycle. After 7 dpi, mosquitoes were fixed in 4% paraformaldehyde overnight at 4 °C. Mosquitoes were mounted in paraffin blocks and sectioned (Colorado Histo-Prep, Fort Collins, CO). In situ hybridization of tissue sections was performed using the ViewRNA ISH 2-plex kit according to manufacturer’s protocol (ThermoFisher, Waltham, MA). NHUV RNA was detected using custom RNA probes targeting conserved regions of the positive-strand RNA. Fixed tissue sections were counterstained with Gills hematoxylin solution (Sigma-Aldrich, St. Louis, MO) and imaged using a Zeiss LSM800 confocal microscope and ZEN software (Carl Zeiss, Oberkochen, Germany).

### Statistics

A two-way ANOVA with an a posteriori Bonferroni correction for multiple comparisons was used to determine differences in viral titers between DENV-2, CHIKV and ZIKV controls and NHUV/DENV-2, NHUV/CHIKV or NHUV/ZIKV dually inoculated groups. Similarly, a two-way ANOVA with an a posteriori Bonferroni correction for multiple comparisons was also used to determine differences in intracellular NHUV and ZIKV copy numbers for C6/36 cells inoculated with ZIKV, NHUV, or NHUV/ZIKV dually inoculated groups. A chi-squared analysis was used to determine differences in infection, dissemination, or transmission between the ZIKV only and ZIKV/NHUV IT inoculated or per orally exposed *Ae. aegypti* mosquito groups.

### Disclaimer

The findings and conclusions in this study are those of the authors and do not necessarily represent the official position of the Centers for Disease Control and Prevention.
